# Biosynthetic Pathway and the Potential Role of Melatonin at Different Abiotic Stressors and Developmental Stages in *Tolypocladium guangdongense*

**DOI:** 10.3389/fmicb.2021.746141

**Published:** 2021-10-08

**Authors:** Gangzheng Wang, Xianglian Chen, Chenghua Zhang, Min Li, Chengyuan Sun, Ning Zhan, Xueshuang Huang, Taihui Li, Wangqiu Deng

**Affiliations:** ^1^Guangdong Provincial Key Laboratory of Microbial Culture Collection and Application, State Key Laboratory of Applied Microbiology Southern China, Institute of Microbiology, Guangdong Academy of Sciences, Guangzhou, China; ^2^Hunan Provincial Key Laboratory for Synthetic Biology of Traditional Chinese Medicine, Hunan University of Medicine, Huaihua, China; ^3^College of Agriculture and Animal Husbandry, Tibet University, Nyingchi, China; ^4^College of Plant Protection, China Agricultural University, Guangzhou, China; ^5^College of Bioscience and Biotechnology, Hunan Agricultural University, Changsha, China

**Keywords:** melatonin, *Tolypocladium guangdongense*, quantification analysis, biosynthetic pathway, resistance to abiotic stressors, primordial formation

## Abstract

Melatonin, a bioactive compound and an important signaling molecule produced in plants and animals, is involved in many biological processes. However, its function and synthetic pathways in fungi are poorly understood. Here, the samples from *Tolypocladium guangdongense*, a highly valued edible fungus with functional food properties, were collected under different experimental conditions to quantify the levels of melatonin and its intermediates. The results showed that the intracellular melatonin content was markedly improved by Congo red (CR), cold, and heat stresses; the levels of intracellular melatonin and its intermediates increased at the primordial (P) and fruiting body (FB) stages. However, the levels of most intermediates exhibited a notable decrease under CR stress. Several genes related to melatonin synthesis, excluding *AADC* (aromatic-L-amino-acid decarboxylase), were markedly upregulated at an early stage of CR stress but downregulated later. Compared to the mycelial stage, those genes were significantly upregulated at the P and FB stages. Additionally, exogenous melatonin promoted resistance to several abiotic stressors and P formation in *T. guangdongense*. This study is the first to report melatonin biosynthesis pathway in macro-fungi. Our results should help in studying the diversity of melatonin function and melatonin-synthesis pathways and provide a new viewpoint for melatonin applications in the edible-medicinal fungus.

## Introduction

Melatonin [N-acetyl-3-(2-aminoethyl)-5-methoxyindole] is an indoleamine that was first reported as a neurohormone in the bovine pineal gland (Lerner et al., 1958). In 1995, it was documented that melatonin can be synthesized in many edible plants, such as tomatoes, bananas, cucumbers, and potatoes (Dubbels et al., 1995; Hattori et al., 1995), which opened the avenues of investigation in melatonin research and attracted the attention of some researchers toward melatonin synthesis in extrapineal tissues. In the last two decades, accumulating evidences has shown that indoleamine is a ubiquitous in many organisms (Balzer and Hardeland, 1996; Hardeland and Poeggeler, 2003; Rodriguez-Naranjo et al., 2012). For fungi, intracellular melatonin was first observed in yeast cells arrested in minimal medium a study conducted by Sprenger et al. (1999). The existence of melatonin has been reported in some macro-fungi, such as *Agaricus bisporus*, *Lentinula edodes*, *Pleurotus ostreatus*, and *Volvaria volvacea* (Muszyńska et al., 2011; Muszyńska and Sułkowska-Ziaja, 2012; Gao et al., 2020).

In 1960, Axelord et al. first reported the classical pathway of melatonin biosynthesis in vertebrates. Using four enzymatic reactions, the precursor tryptophan was successively converted into 5-hydroxytryptophan (5HTryp), serotonin, and N-acetylserotonin (NAS) as intermediates (Axelrod and Weissbach, 1960). The melatonin-synthesis pathway in plants differs from that in vertebrates (Arnao and Hernández-Ruiz, 2017). In plants, tryptophan is first converted into tryptamine by tryptophan decarboxylase (TDC) or into 5HTryp by tryptophan 5-hydroxylase (TPH). Second, tryptamine is catalyzed into N-acetyltryptamine (NAT) and 5HT *via* serotonin N-acetyltransferase (SNAT) and tryptamine 5-hydroxylase (T5H), respectively, whereas 5HTryp is converted into serotonin by TDC. Third, T5H and SNAT catalyze the conversion of NAT and 5HT, respectively, into N-acetylserotonin (NAS), whereas serotonin is converted into 5-methoxytryptamine (5MT) by acetylserotonin O-methyltransferase (ASMT) or caffeic acid O-methyltransferase (COMT). Finally, NAS and 5MT are converted into melatonin by ASMT and SNAT, respectively. Muñiz-Calvo et al. (2019) analyzed the melatonin-synthesis pathway in *Saccharomyces cerevisiae* by quantifying changes in the levels of melatonin and its biosynthetic intermediates in yeast cells at different growth stages. The synthesis pathway in yeast was similar to that in plants, except that NAT were not detected. Nevertheless, the melatonin-synthesis pathway has not been elucidated in other fungi.

Melatonin plays an extremely important role as a multifunctional signaling molecule during responses to physiochemical processes. In animals and humans, melatonin has been shown to be mainly involved in photoperiodism and circadian rhythms and play various biological roles such as immunomodulatory, anticancer, anti-aging, and anti-obesity effects (Motilva et al., 2011; Salehi et al., 2019; Moradkhani et al., 2020). In plants, melatonin has shown many beneficial effects in term of seed germination, growth promotion, yield improvement, fruit preservation, and resistance against biotic and abiotic stresses (Posmyk et al., 2008; Wei et al., 2014; Aghdam et al., 2020; Nawaz et al., 2021). In *Saccharomyces* and non-conventional yeasts, melatonin can alleviate the damage induced by oxidative stress and ultraviolet stress (Vázquez et al., 2017, 2018; Bisquert et al., 2018). In addition, melatonin can regulate the fermentative capacity of yeasts by interacting with glycolytic proteins (Morcillo-Parra et al., 2020b). Several recent studies have reported that exogenous melatonin enhanced the cadmium tolerance in *V. volvacea* and retarded the senescence and the degree of cap browning in *A. bisporus* during the storage process (Gao et al., 2020; Li et al., 2021). In addition, limited information is available regarding the effects of melatonin on fungi.

*Tolypocladium guangdongense*, also named as *Cordyceps guangdongensis*, belongs to the same family as the traditional Chinese medicine *Ophiocordyceps sinensis*. The *in vivo* studies has shown that *T. guangdongense* markedly affected the growth of the influenza virus H9N2, reducing anti-inflammation in chronic bronchitis and alleviating chronic renal failure (Yan et al., 2010, 2012, 2014). These results indicate that *T. guangdongense* has the potential as an important economic fungus and can be applied in the functional food and healthcare industries. In addition, in our recent study, tryptophan and tryptamine levels peaked at the primordial (P) stage (Wang et al., 2020a), and we detected melatonin and several intermediates when performing the metabolome analysis of *T. guangdongense* fruiting bodies (unpublished data), suggesting that the levels of melatonin and the associated intermediates notably differ during the growth and development process of *T. guangdongense*.

The aims of the current study were to quantify the levels of melatonin and its intermediates in *T. guangdongense* under abiotic stress [due to Congo red (CR), H_2_O_2_, heat, and cold stresses] and during different developmental stages. We also sought to identify the biosynthetic pathway of melatonin in *T. guangdongense* and analyzed the expression pattern of genes related to melatonin biosynthesis under different experimental conditions. Furthermore, we aimed to explore the potential role of melatonin in response to abiotic stress and in primordial formation. These results show the diversity of melatonin-synthesis pathways and its function and provide an important guiding significance of melatonin applications in the edible-medicinal fungus.

## Materials and Methods

### Fungal Material and Treatments

*Tolypocladium guangdongense* strain CCTCCM206051 was used in this study and deposited in the Agricultural Culture Collection of China. Several mycelial plugs were inoculated into 200ml improved CYM medium (20g dextrose, 2g yeast extract, 2g peptone, 1g KH_2_PO_4_, 1g NaNO_3_, 0.5g K_2_HPO_4_, 0.46g MgSO_4_•7H_2_O, and 0.1g vitamin B1 in 1l ddH_2_O), and cultured for 10days in an incubator shaker at 140 rotations/min (rpm) and 23°C. Next, mycelial pellets were transferred to PDA (potato dextrose agar) medium with or without 10μM H_2_O_2_ and 2mg/ml CR and incubated for 14days at 23°C. Mycelia cultured at 23°C for 4days were subjected to 14days 30°C or 14°C treatment as heat treatment (HT) and cold treatment (CT), respectively. In addition, the samples of mycelium (My), P, and young fruiting body (FB) were collected as described previously (Wang et al., 2020a). All the samples were immediately frozen in liquid nitrogen and then were preserved at −80°C until metabolite, and RNA isolation were performed.

### Quantification of Intracellular Melatonin Levels

Samples (0.1g) from different experimental conditions were homogenized in 1ml of a methanol/water (50:50, v/v) mixture with 1.0ng/g [^2^H_4_] MLT as an internal standard. After vortex-induced vibration for 15min, the mixture was centrifuged at 12,000rpm for 5min at 4°C, and the supernatant was collected. This procedure was repeated once. Both supernatants were merged and evaporated under a mild nitrogen stream. Then, the evaporated samples were re-dissolved in 100μl methanol/water (50:50, v/v) mixture. After 5min vortex-induced vibration, the mixture was centrifuged at 12,000rpm for 5min and filtered using a 0.22μm filter membrane. Intracellular melatonin levels were quantified by Wuhan Metware Biotechnology Co., Ltd. (Wuhan, China) using an liquid chromatography–tandem mass spectrometry (LC–MS/MS) platform (Waters Iclass-AB Sciex QTRAP 6500+) platform as described by Ye et al. (2017).

### Qualification of Levels of Indolic Compounds Related to Melatonin Biosynthesis

The indolic compounds tryptophan, tryptamine, 5HTryp, NAT, serotonin, NAS, and 5MT were purchased from Sigma-Aldrich (Madrid, Spain) and Shanghai Yuanye Bio-Technology Co., Ltd (China). These indolic compounds were extracted using the method described in our previous study (Wang et al., 2020a). Briefly, sample tissues (0.5g) from the CR and control (CK) groups was finely ground, followed by extraction in an 1ml buffer containing 50% methanol and 0.1% formic acid for 15min in a shaker. After ultrasound treatment for 30min in an ice bath, the mixtures were incubated at 4°C for 30min and then centrifuged for 20min at 12,000rpm. The extracts were filtered through a 0.3μm filter membrane and were analyzed using LC–MS/MS platform (Waters Iclass-AB Sciex 6500) platform at Beijing Genomics Institute (BGI)-Shenzhen (Wuhan, China) as described by Sara et al. (2019).

### Expression Patterns of Genes Related to Melatonin Biosynthesis Under Different Conditions

Genes related to melatonin biosynthesis in *T. guangdongense* were predicted by comparing the amino acid sequences with those of *Oryza sativa* and *Mus musculus* in *T. guangdongense* genome (BioProject: PRJNA399600). Tissue samples collected from My, P, and FB stages and the different periods of CR stress (6h, 12h, 1days, 2days, 4days, 8days, and 14days) and were used for RNA isolation using the OminiPlant RNA Kit (DNase I; CWBIO, Beijing, China). The mRNA was reverse transcribed into cDNA and used for quantitative reverse transcriptase-polymerase chain reaction (qRT-PCR) analysis of genes related to melatonin biosynthesis. The gene-specific primers used for gene quantification were designed using Primer Primer5.0, and their sequences were shown in Table_S1. Histone *H4* and the vacuolar protein sorting gene *VPS* were used as the internal control genes. The qRT-PCR reaction was performed using an Applied Biosystems ABI 7500 instrument (ABI, Foster City, California, United States) and three biological and technical repeats. PCR reaction was performed with an initial denaturation for 3min at 95°C, followed by 40cycles of 10s at 95°C and annealing at 60°C for 20s. The relative expression levels of the target genes were calculated using the 2^−△△Ct^ method (Wang et al., 2020b).

### Effects of Exogenous Melatonin on *Tolypocladium guangdongense* Under Different Conditions

Ten micrometer H_2_O_2_, 2mg/ml CR, and 10μM melatonin were added to PDA medium at approximately 55°C. Mycelial pellets activated for 10days were inoculated into PDA medium and cultured at 23°C. For the HT and CT groups, the PDA plates containing 10μM melatonin were placed in an incubator at 30°C and 14°C. After 24days of culture, the diameters of the fungal colonies were measured to assess the effects of melatonin on *T. guangdongense* mycelia under different experimental conditions. In addition, the groups containing 10μM melatonin were transferred into the cultivation room at a temperature of approximately 23°C and relative humidity of 60–70%. Meanwhile, the mycelial pellets were subjected to treatment of 10μM melatonin, and then were inoculated in rice medium. After 15days culture, they were transferred into the cultivation room at a temperature of approximately 23°C and relative humidity of 60–70%. The mycelia were illuminated (500lx) for 10h each day to explore the effect of melatonin on primordial formation.

### Statistical Analysis

Significant differences were determined using the Statistical Package for the Social Sciences program (SPSS 22.0, SPSS Inc., Chicago, IL, United States). Error bars represent as the standard deviation from the mean of 10 independent replications in every group. Lowercase letters and asterisks were used to refer to significant differences between each group and the control group (CK).

## Results and Discussion

### Experimental Factors Affected Production of Intracellular Melatonin

The levels of endogenous melatonin are regulated by multiple environmental factors. For example, endogenous melatonin synthesis was promoted by salt stress and a high temperature in sunflower and *Arabidopsis* seedlings (Mukherjee et al., 2014; Shi et al., 2015). In *S. cerevisiae*, a low temperature and low sugar level delayed the peaking of intracellular melatonin levels, whereas melatonin synthesis production was significantly promoted at 12°C (Wang et al., 2016; Morcillo-Parra et al., 2020a). To determine whether the intracellular melatonin levels of *T. guangdongense* markedly differ under different experimental conditions, *T. guangdongense* samples from three different developmental stages and four different abiotic stresses were collected, and melatonin contents were measured by LC–MS/MS.

**Figure 1:**
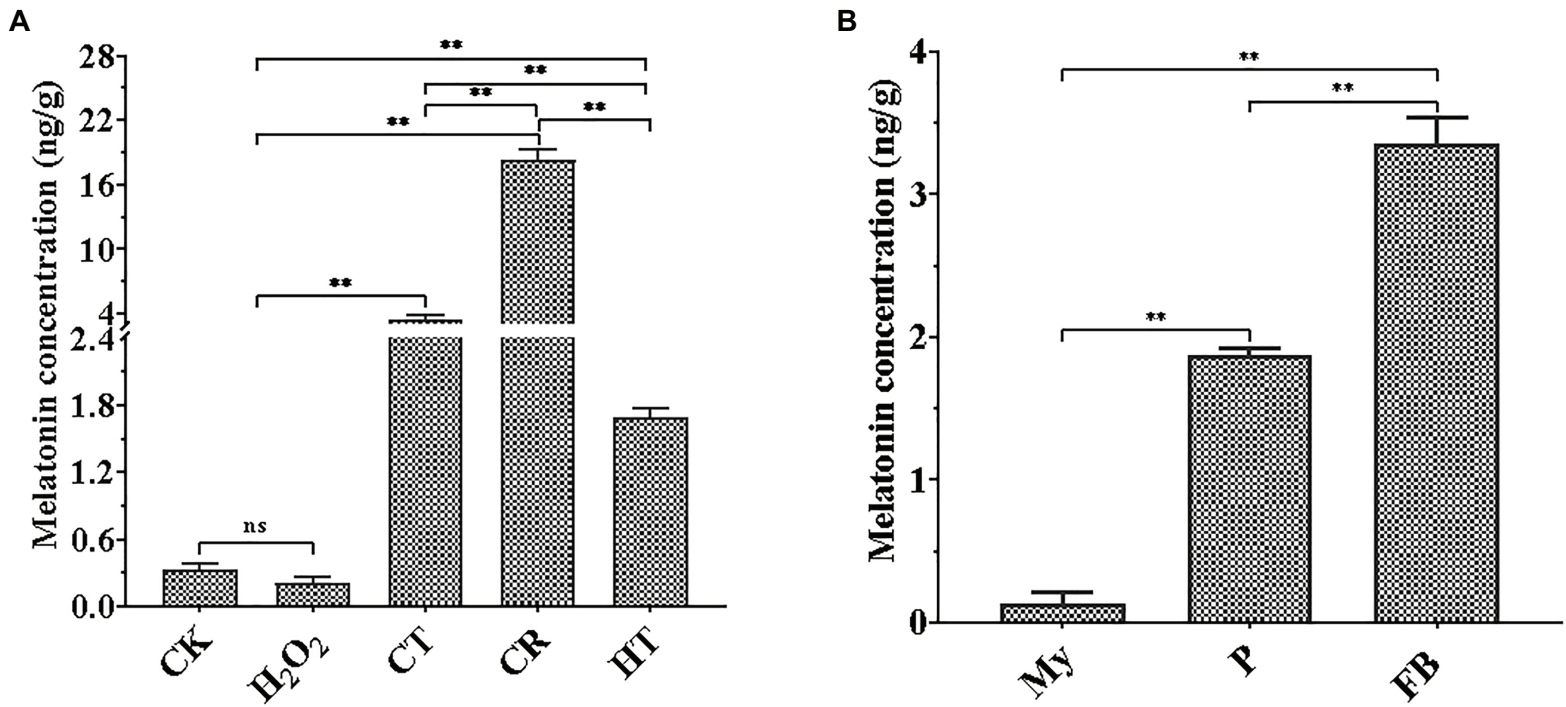
Quantification of melatonin production in *T. guangdongense* at different abiotic stressors **(A)** and developmental stages **(B)**. CK, mycelia untreated by several abiotic stresses; H_2_O_2_, H_2_O_2_ stress; CT, cold stress; CR, congo red stress; My, mycelial stage; HT, heat stress; P, primordial stage; FB, fruiting body stage; **, highly significant differences (*p*<0.01).

As shown in Figure 1A, three types of abiotic stresses significantly promoted intracellular melatonin production, whereas H_2_O_2_ stress tended to a decrease trend in intracellular melatonin synthesis. Compared to the intracellular melatonin content of the CK, CR exhibited the highest increase level (approximately 55.6-fold), flowed by CT (approximately 10.6-fold), and HT (approximately 5.13-fold), which was similar to the increase of melatonin levels in *V. volvacea* under Cd stress and *A. bisporus* during low temperature storage (Aghdam et al., 2019; Gao et al., 2020). Compared to My stage, intracellular melatonin levels had approximate 16-fold and 28-fold increase at the P and FB stages, respectively (Figure 1B). These results suggested that intracellular melatonin may be involved in the primordial formation, fruiting body growth and the response of *T. guangdongense* to abiotic stresses, which need to be confirmed by further study.

### Analysis of Intermediates Levels and Expression Pattern of Genes Related to Melatonin Synthesis Under Different Experimental Conditions

In *S. cerevisiae*, Muñiz-Calvo et al. (2019) deciphered the melatonin biosynthesis and metabolism based on the bioconversion of related metabolites. According to their results and metabolome analysis conducted in our laboratory, the intermediates related to melatonin biosynthesis were detected in the mycelia and fruiting bodies of *T. guangdongense* (unpublished data). To more accurately quantify their dynamic changes and identify the melatonin-synthesis pathway, biosynthetic intermediate levels were quantified in fungal samples collected after 14days CR stress and during different developmental stages. The amino acid sequences of proteins related to melatonin biosynthesis in *O. sativa* and *M. musculus* were characterized by BLAST analysis against the genome of *T. guagndongense*. Proteins with an E-value of <0.001 and >35% amino acid similarity were treated as the homologous proteins. In the genome of *T. guangdongense*, nine genes were predicted to be the homologous genes involved in melatonin biosynthesis (Supplementary Table S1).

**Figure 2:**
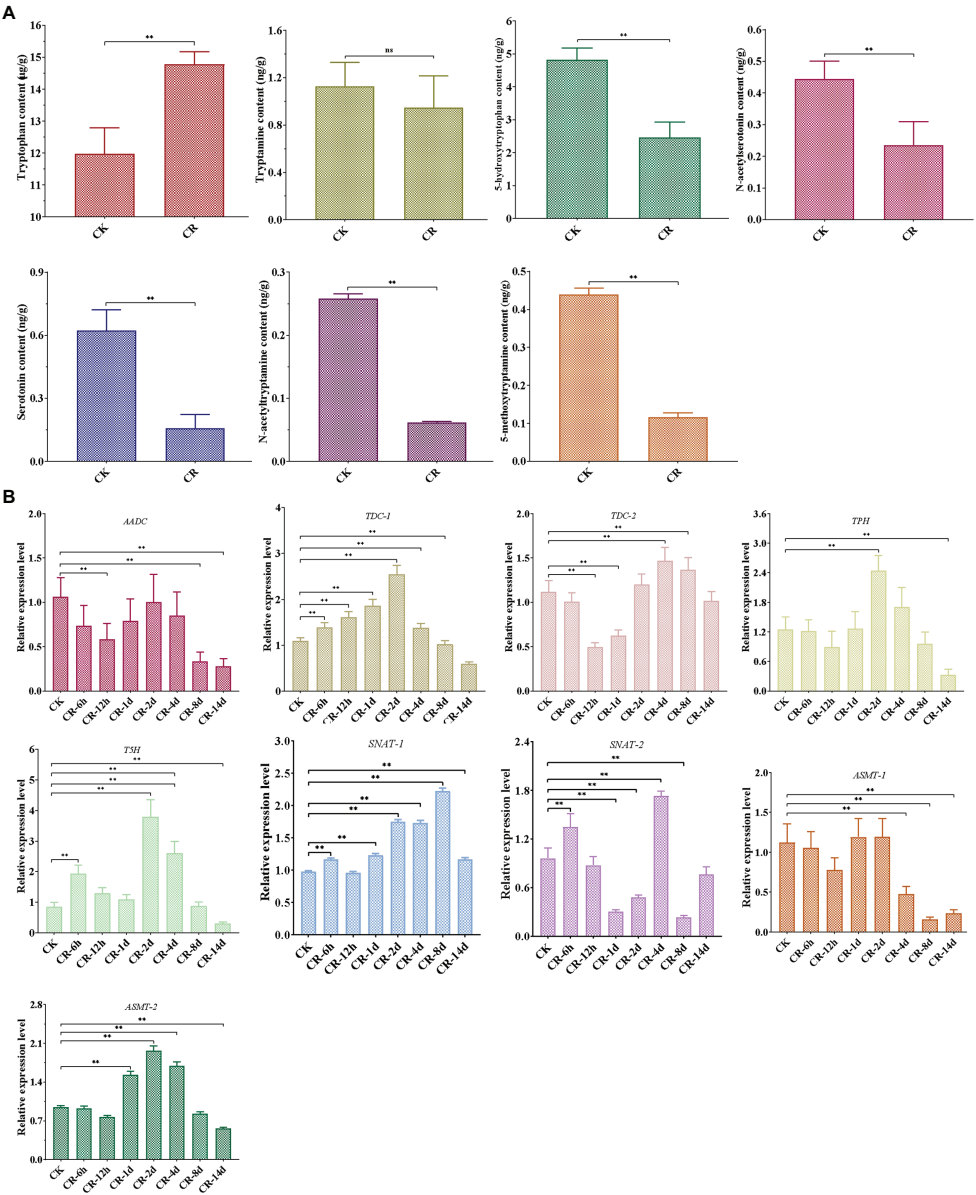
Detection of melatonin-biosynthesis intermediates after 14 d CR treatment **(A)** and expression patterns of genes related to melatonin synthesis at different periods of CR stress **(B)** in *T*. *guangdongense*. CK, group at 0h.; CR, congo red; *AADC*, aromatic-L-amino-acid decarboxylase; *TDC*, tryptophan decarboxylase; *TPH*, tryptophan 5-hydroxylase; *T5H*, tryptamine 5-hydroxylase; *SNAT*, serotonin N-acetyltransferase; *ASMT*, acetylserotonin O-methyltransferase; ns, no significant difference. **, highly significant differences (*p*<0.01).

Our LC–MS/MS analysis revealed that the abundance of most intermediates markedly changed under CR condition compared to the corresponding levels found under normal culture condition (Figure 2A). The levels of tryptophan, the precursor of melatonin, were significantly increased in the CR medium. Tryptamine levels were not significantly different in the CR and CK media; however, the levels of four other intermediates exhibited a remarkable decease under CR condition. This phenomenon was inconsistent with the significant increase of melatonin content detected after being subjected to 14days of CR stress. Therefore, the relative levels of these genes related to melatonin synthesis were analyzed during different periods of CR stress. As shown in Figure 2B, six genes were markedly upregulated in the early period but significantly downregulated in the late period, whereas the relative expression level of two genes showed an obvious down-regulation on days 8 and 14. Specifically, five genes (*TDC-1*, *TPH*, *T5H*, *SNAT-1*, and *ASMT-2*) were significantly upregulated during the first 8days of CR stress, but markedly downregulated on day 14. The relative expression levels of *AADC* and *ASMT-1* decreased significantly on days 8 and 14. *TDC-2* was markedly upregulated on day 8 but downregulated after 12h and on day 1. *SNAT-2* was significantly downregulated on days 1, 2, and 8, whereas was markedly upregulted after 6h and on day 4. From the perspective of gene expression pattern at different periods of CR stress, we speculated that the intermediates were synthesized and began to accumulate on day 8 or during an earlier period of CR stress, resulting in the accumulation and increase of melatonin at 14 d of CR stress.

**Figure 3:**
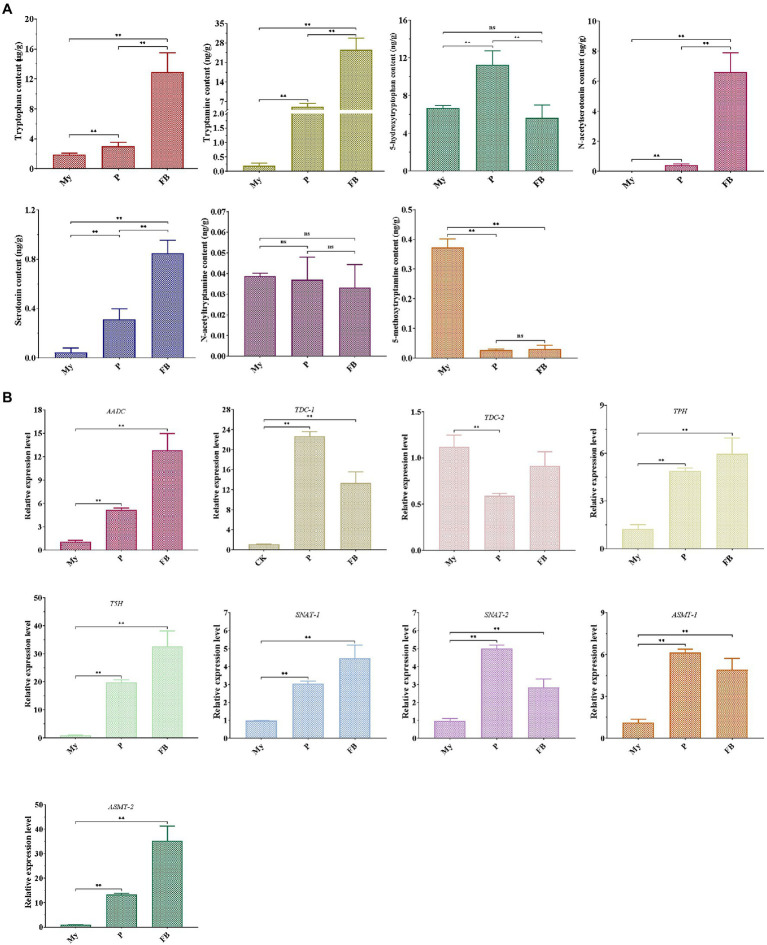
Detection of melatonin-biosynthesis intermediates **(A)** and the expression pattern of genes related to melatonin synthesis **(B)** at different development stages of *T. guangdongense*. My, mycelial stage; P, primordial stage; FB, fruiting body stage; *AADC*, aromatic-L-amino-acid decarboxylase; *TDC*, tryptophan decarboxylase; *TPH*, tryptophan 5-hydroxylase; *T5H*, tryptamine 5-hydroxylase; *SNAT*, serotonin N-acetyltransferase; *ASMT*, acetylserotonin O-methyltransferase; ns, no significant difference. **, highly significant differences (*p*<0.01).

LC–MS/MS analysis showed that compared to My stage, the levels of five metabolites (tryptophan, tryptamine, 5HTryp, serotonin, and NAS) were significantly increased at the P and FB stages, whereas the levels of 5MT were markedly reduced (Figure 3A). In terms of the expression profile of genes related to melatonin synthesis, all the genes were significantly upregulated at P and FB stages compared to My stage (Figure 3B), being consistent to the higher levels of the intermediates at the P and FB stages. Comparing gene expression levels at the P and FB stages, the relative levels of six genes (*AADC*, *TDC-2*, *TPH*, *T5H*, *SNAT-1*, and *ASMT-2*) showed a significant upregulation at the FB stage, and two genes (*TDC-1* and *SNAT-2*) were markedly upregulated at the P stage, in agreement with the quantitative analysis of the intermediate levels.

**Figure 4:**
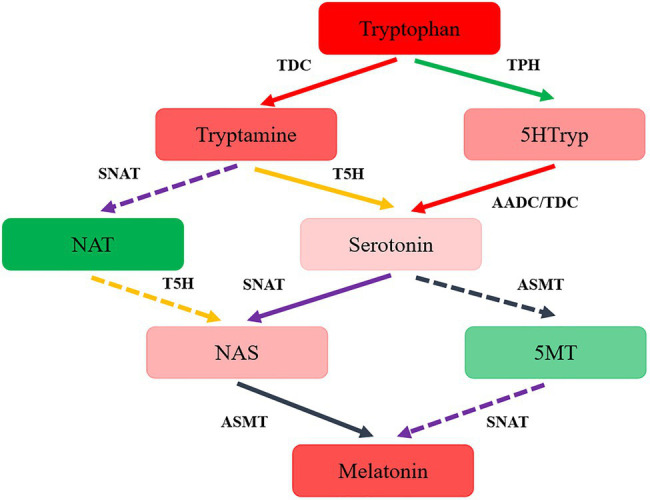
The hypothesized pathway of melatonin biosynthesis in *T. guangdongense*. 5HTryp, 5-Hydroxytryptophan; NAT, N-acetyltryptamine; NAS, N-acetylserotonin; 5MT, 5-methoxytryptamine; AADC, aromatic-L-amino-acid decarboxylase; TDC, tryptophan decarboxylase; TPH, tryptophan 5-hydroxylase; T5H, tryptamine 5-hydroxylase; SNAT, serotonin N-acetyltransferase; ASMT, acetylserotonin O-methyltransferase; Full line represent the main procedures; colors, the higher degrees of red or green indicate the more or less levels of the intermediate contents, respectively.

The synthesis pathway of melatonin was speculated in *T. guangdongense*, based on the above results and the related results from yeast and plants (Muñiz-Calvo et al., 2019; Nawaz et al., 2021). As shown in Figure 4, the putative melatonin-biosynthesis pathway in *T. guangdongense* was relatively complex compared to that in *S. cerevisiae*. In the first step of the proposed pathway, tryptophan was converted into tryptamine and 5HTryp by TDC/AADC and TPH, respectively. Later, this tryptamine was converted to NAT or serotonin by acetyltation (SNAT) and hydroxylation (T5H), respectively, whereas 5HTryp was converted to serotonin *via* 5-hydroxytryptophan carboxylation (TDC/AADC). Subsequently, serotonin was converted to NAS and 5MT by N-acetylation (SNAT) and O-methylation (ASMT), respectively, whereas NAT was hydroxylated by T5H to form NAS. Finally, *T. guangdongense* can covert NAS and five MT into melatonin by O-methylation (ASMT) and N-acetylation (SNAT), respectively. According to the above results, it was suggested that serotonin and NAS were the main melatonin-synthesis intermediates, and the melatonin-biosynthesis pathway in *T. guangdongense* was markedly similar to that in plants (Nawaz et al., 2021).

### Exogenous Melatonin Promoted Mycelial Growth of *Tolypocladium guangdongense* Under Abiotic Stress Conditions

By interacting with other signaling molecules, such as reactive oxygen species and nitric oxide, melatonin participates in various physiological reactions and enhances the resistance of plants to abiotic stress (Yu et al., 2018; Qiao et al., 2019). In fungi, exogenous melatonin enhanced the resistance of *S. cerevisiae* and *V. volvacea* against oxidative stress and cadmium stress, respectively (Vázquez et al., 2017; Gao et al., 2020). Therefore, colony diameters under different experimental conditions were measured to evaluate the effect of exogenous melatonin on *T. guangdongense* mycelia.

**Figure 5:**
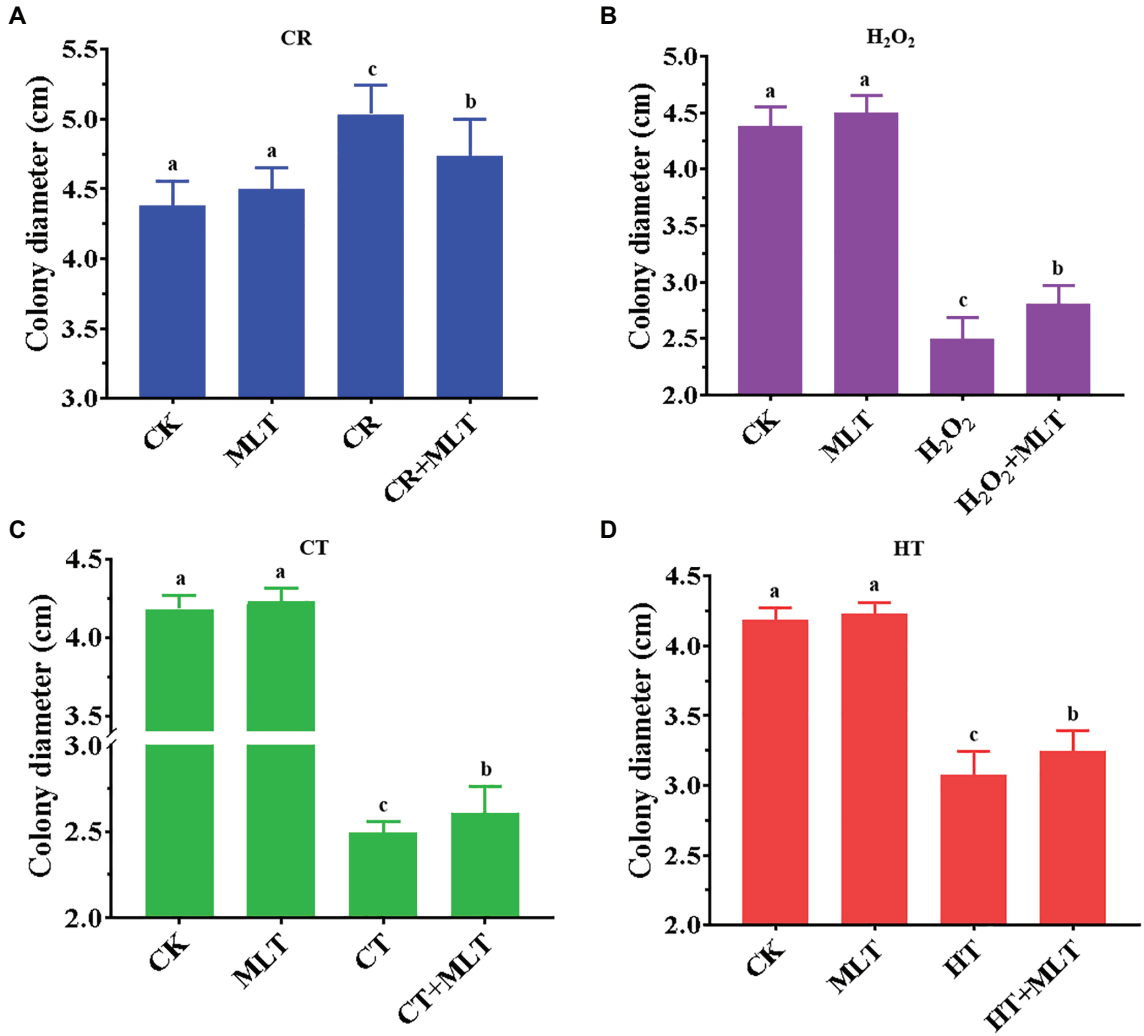
Effect of exogenous melatonin on the mycelial growth of *T. guangdongense* under CR **(A)**, H2O2 **(B)**, CT **(C)** and HT stress **(D)**, respectively. CT, cold stress; CR, cango red; HT, heat stress; MLT, melatonin; Lowercase letters represent significant difference (*p*<0.05).

Previously, it was found that treatment with 1μM and 1M melatonin obviously inhibited the mycelial growth of *T. guangdongense*, whereas no significant difference was found after treatment with 10μM or 100μM melatonin (Supplementary Figure S1). In addition, the mean of colony diameters in the 10μM melatonin group was the largest (4.23cm). Therefore, 10μM melatonin was selected for this study. In terms of the four abiotic stresses applied, 2mg/ml CR showed an evident promotion of the mycelial growth, whereas the other three stresses had a significantly negative effect on the mycelial growth of *T. guangdongens*e (Figure 5). The stimulatory effect of 2mg/ml CR was reduced in the presence of exogenous melatonin (Figure 5A). Nevertheless, exogenous melatonin promoted the mycelial growth of *T. guangdongense* and enhanced its resistance to heat, cold, and H_2_O_2_ stresses (Figures 5B-D), which was consistent with previous results (Wei et al., 2014; Yu et al., 2018; Nawaz et al., 2021). These results suggested that melatonin plays important roles in the response of *T. guangdongense* to abiotic stresses.

### Exogenous Melatonin Promoted Primordial Formation of *Tolypocladium guangdongense*

**Figure 6:**
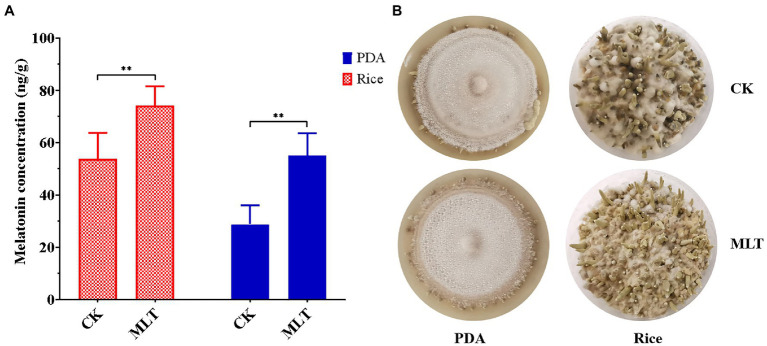
Effect of exogenous melatonin on the primordial formation in *T. guangdongense*. (**A)** Primordial numbers of *T. guangdongense* on PDA and rice medium, respectively. (**B)** Primordial characteristics of *T. guangdongense* on PDA and rice medium. CK, group untreated by melatonin; MLT, group treated by 10μM melatonin. **, highly significant differences (*p*<0.01).

As a growth-stimulating compound, melatonin is involved in several biological functions in plants such as the growth of roots and coleoptiles, seed germination, seedling growth, and senescence (Hernández-Ruiz et al., 2005; Wang et al., 2013; Arnao and Hernández-Ruiz, 2019). In this study, it was found that intracellular melatonin levels obviously increased at the P and FB stages (Figure 1B). Therefore, exogenous melatonin was added to PDA and rice medium to evaluate the effect of melatonin on primordial formation. Significantly more primordia were observed in the melatonin treatment group than those in the control group (Figure 6A). Additionally, the primordial density had an approximately one-fold increase in the melatonin treatment group (Figure 6B), indicating an important regulatory role in primordial formation in *T. guangdongense*. These results suggested that exogenous melatonin has the good potential for enhancing the cultivation of edible-medicinal mushroom.

## Conclusion

In this study, we first quantified melatonin and the related biosynthetic intermediates in the macro-fungi. Several abiotic stressors promoted the synthesis of melatonin in *T. guangdongense*, and the melatonin and intermediate contents were relatively higher at the P and FB stages. However, the levels of most intermediates showed an obvious decrease after 14days CR stress. After different periods of CR stress, most genes related to melatonin biosynthesis were markedly upregulated in the earlier period, but downregulated in the latter period, yet their relative expression levels showed a significant upregulation at the P and FB stages. On the basis of these results, a model of the melatonin-synthesis pathway was proposed in *T. guangdongense*. In addition, exogenous melatonin enhanced the resistance of *T. guangdongense* to the abiotic stress and promoted primordial formation. However, the underlying regulatory mechanism of melatonin remains unknown. In future study, it will be necessary to determine whether melatonin regulates the growth and development of fruiting bodies and the biosynthesis of active ingredients. In addition, molecular mechanisms regulating development, resistance to stress, and metabolite synthesis merit a comprehensive exploration.

## Data Availability Statement

The original contributions presented in the study are included in the article/Supplementary Material, further inquiries can be directed to the corresponding authors.

## Author Contributions

GW, CZ, TL, and WD conceptualized the study. GW and WD helped with the data curation. GW, XC, ML, and NZ contributed to the formal analysis. XH, TL, and WD were responsible for the funding acquisition. ML, CS, and XC carried out the investigation. GW, CZ, XC, ML, CS, and WD worked on the methodology. TL contributed to the resources. GW, CS, and XC were responsible for the validation. GW performed the visualization and wrote the original draft. XH, TL, XC, and WD reviewed and edited the manuscript. All authors contributed to the article and approved the submitted version.

## Funding

This research was funded by the National Natural Science Foundation of China (grant no. 31900015 and 31970024), Hunan Provincial Key Laboratory for Synthetic Biology of Traditional Chinese Medicine (no. HCSW2020-02), the Science and Technology Planning Project of Guangdong Province, China (nos. 2019B121202005), and the GDAS’ Special Project of Science and Technology Development (no. 2020GDASYL-20200103014).

## Conflict of Interest

The authors declare that the research was conducted in the absence of any commercial or financial relationships that could be construed as a potential conflict of interest.

## Publisher’s Note

Publisher’s NoteAll claims expressed in this article are solely those of the authors and do not necessarily represent those of their affiliated organizations, or those of the publisher, the editors and the reviewers. Any product that may be evaluated in this article, or claim that may be made by its manufacturer, is not guaranteed or endorsed by the publisher.

## Supplementary Material

Supplementary Figure S1 Effect of different concentration melatonin on the mycelial growth of *T. guangdongense*.

Supplementary Table S1 Information of genes related to melatonin biosynthesis.
