# Supplementary figures and images for: Biosynthetic Pathway and the Potential Role of Melatonin at Different Abiotic Stressors and Developmental Stages in Tolypocladium guangdongense
Source: Front Microbiol. 2021 Oct 8;12:746141. doi: 10.3389/fmicb.2021.746141 (PMC8533646; doi:10.3389/fmicb.2021.746141)

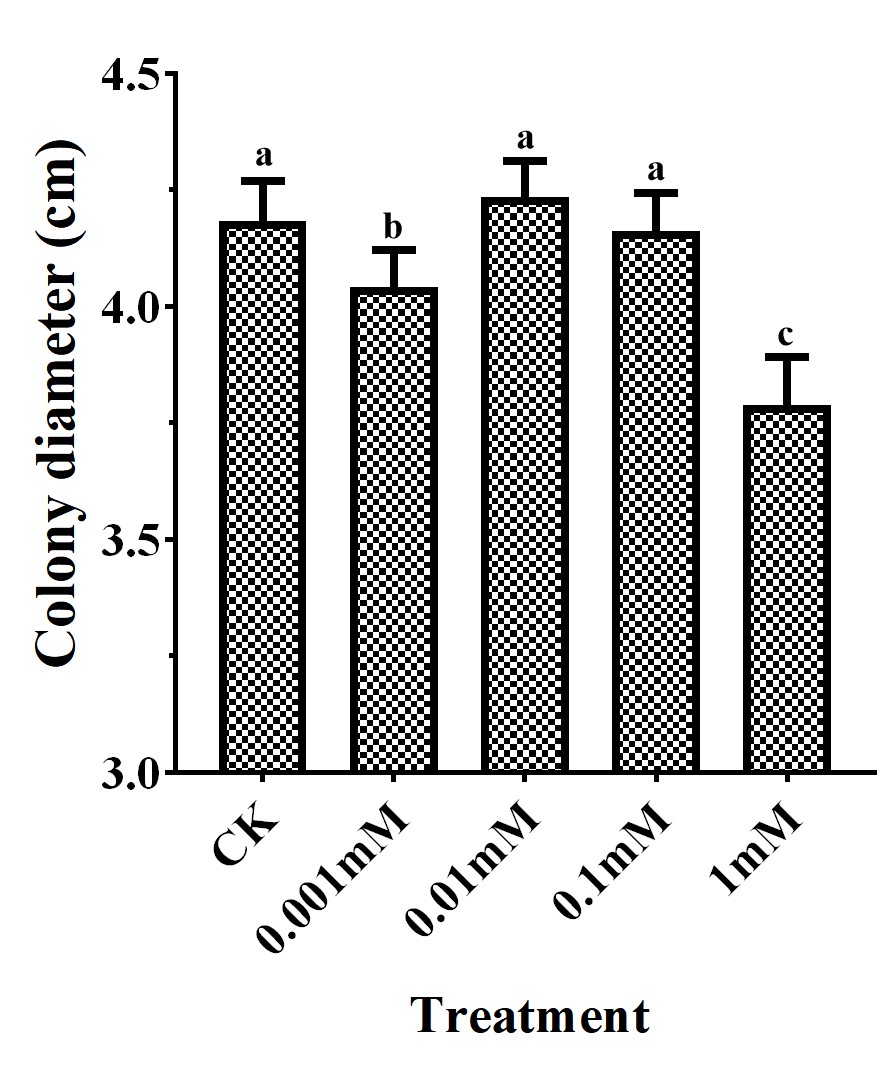

Supplement: Supplementary Table S1 — Information of genes related to melatonin biosynthesis. [file Image_1.JPEG]
